# Glycated Haemoglobin (HbA1c) Variations in Nondiabetics With Nutritional Anemia

**DOI:** 10.7759/cureus.11479

**Published:** 2020-11-13

**Authors:** Rakesh Pilla, Sujith Kumar Palleti, Renuka Rayana, Satish Reddy SKSS, Aminah Abdul Razzack, Sruti Kalla

**Affiliations:** 1 Internal Medicine, Maharajah's Institute of Medical Sciences, Vizianagaram, IND; 2 Internal Medicine, MNR Medical College & Hospital, Sangareddy, IND; 3 Internal Medicine, Andhra Medical College, Visakhapatnam, IND; 4 Internal Medicine, Mallareddy Medical College For Women, Hyderabad, IND

**Keywords:** anemia, iron deficiency, b12 deficiency, hba1c

## Abstract

Objectives:

Diabetes is prevalent in the Indian population, to the extent that the diabetes burden matches that of nutritional anemia. We aimed to determine the effects of iron and vitamin B12 deficiency anemia on glycated haemoglobin (HbA1c) concentrations in individuals without diabetes.

Material and methods:

The study comprises 100 patients with iron deficiency anemia, 100 with vitamin B12 deficiency anemia, and 100 healthy volunteers as a control group. Each of the first two groups was subdivided into two groups depending on the severity of anemia based on Hb levels. We treated with iron replenishment in the iron deficiency group and B12 replenishment in the B12 deficiency group for three months. We noted HbA1c levels before and after the therapy. Data were entered into the SPSS package. For comparing pre and post-therapy levels, we used the Paired 't' test.

Results:

The mean HbA1c before treatment were 6.1% ± 0.23% and 5.5% ± 0.24%, and the values after treatment were 5.1% ± 0.14% and 4.6% ± 0.2% in severe iron deficiency anemia subgroup and mild to moderate subgroup, respectively. The mean HbA1c in the iron-deficiency anemia control group was 5.2% ± 0.2%. The mean HbA1c levels before treatment were 5.9% ± 0.3% and 5.6% ± 0.19%, and after treatment were 5.0% ± 0.15% and 4.9% ± 0.16% in severe and mild to moderate B12 deficiency anemia, respectively. The mean HbA1c in the vitamin B12 deficiency anemia control group was 5.1% ± 0.2%.

Conclusion:

HbA1c in both types of anemia subjects showed a significant decrease with appropriate therapy. Physicians should consider rechecking patient haemoglobin values and correcting a patient's anemia before determining the patient's glycemic status using HbA1c to avoid misinterpretation of their diabetes status.

## Introduction

Glycated haemoglobin (HbA1c) is defined as haemoglobin (Hb) that is irreversibly glycated at one or both N-terminal valines of the beta chains and remains in the red blood cell (RBC) for the rest of its lifespan [1,2]. Therefore, HbA1c is used as a measure of a patient's glycemic status over the past three months [3]. HbA1c concentrations are not only altered by blood glucose levels [4], but they also vary significantly in a few diseases and pathological states, such as hypoproliferative anemia and hemoglobinopathies [5,6].

Conditions that prolong the erythrocyte lifespan or conditions related to decreased RBC turnover will lead to prolonged exposure of the cell to glucose, resulting in higher HbA1c [7]. Similarly, states that reduce the lifespan of RBCs or conditions where RBC exposure to glucose is shortened (i.e., from an increase in RBC turnover) will result in reduced HbA1c concentrations. Iron deficiency anemia and vitamin B12 deficiency anemia fall into the former scenario.

Initially, we considered HbA1c as a marker for monitoring glycemic control. However, recently HbA1c has also been recommended as a diagnostic indicator of diabetes [5]. The American Diabetes Association (ADA) included HbA1c alone as a diagnostic tool for diabetes in 2010 [1], and we still consider HbA1c a reliable marker for diabetes control as per the 2018 ADA guidelines [8]. Factors altering HbA1c concentrations are Hb variants [9], hemolytic anemia, renal failure [10], hypothyroid [11], alcoholism, and hyperbilirubinemia. In this study, we aimed to look for HbA1c variations in nutritional anemia and their consideration with HbA1c as a diagnostic tool.

Our aims and objectives were to determine the effects of iron and vitamin B12 deficiency anemia on HbA1c in individuals without diabetes, to study HbA1c in anemia patients, and to study how variations in HbA1c may correlate to the degree of anemia.

## Materials and methods

This was a prospective follow-up study consisting of 300 subjects (100 iron-deficient patients, 100 B12-deficient anemia patients, and 100 healthy controls). The study was performed at the Maharajah Institute of Medical Sciences from December 2017 to November 2019.

The ethical committee gave ethical clearance for the present study (MIMS/IEC - 11/2017). The 300 subjects were included after providing written and informed consent. History and physical examination data were thoroughly collected to rule out other causes of anemia, and investigations including Hb (%) values, serum iron, serum ferritin, serum B12 values, fasting blood glucose (FBG) levels, and HbA1c concentrations were conducted.

Our study included iron and or B12 deficiency anemia patients aged 18 years to 70 years of either sex and willing to undergo required investigations. Our study excluded patients with hemolytic anemia and aplastic anemia, patients with diabetes mellitus, pregnant women, and patients with anemia of chronic diseases.

The study population was selected per our inclusion criteria, and the controls were picked to match the gender and age of the study groups. The study population of both iron deficiency anemia and vitamin B12 deficiency anemia were further divided into two groups depending on the degree of anemia. Group 1 included patients with severe anemia (i.e., Hb < 8 g/dL). Group 2 consisted of patients with mild and moderate anemia (i.e., Hb 8 to 11.9 g/dL in women, Hb 8 to 12.9 g/dL in men per the World Health Organization 2011 guidelines [12]). Their Hb%, serum iron, ferritin, B12 values, and HbA1c were recorded, and they were treated with iron replenishment in the iron deficiency group and B12 replenishment in the B12 deficiency group for three months. The same investigations were repeated in follow-up visits until the anemia was corrected, and final values were recorded for our study. The change in HbA1c concerning the correction of each of these anemia types were recorded.

For statistical analysis, data were entered into SPSS for Windows, Version 16.0. (Chicago, SPSS Inc.). For comparing parameters before and after treatment, we used the paired t-test. We calculated mean Hb and mean HbA1c for each type of anemia. The standard deviation was calculated, and the p-value was obtained. A p-value < .05 was considered statistically significant.

## Results

The mean age of patients with iron deficiency anemia was 38 years, whereas patients in the vitamin B12 deficiency anemia had a mean age of 45 years. The mean age of the control group patients was 40 years. The mean FBG levels in the iron-deficiency anemia group were 86 ± 5.72 g/dL, the mean FBG in the vitamin B12 deficiency group was 90 ± 3.12 g/dL, the mean FBG in the control group was 80 ± 8.6 g/dL.

Regarding the correction of the underlying anemia, the mean Hb levels (g/dL) among the iron-deficiency anemia groups before and after treatment are presented in table 1. Table 2 presents the mean Hb levels (g/dL) among vitamin B12 deficiency anemia groups before and after treatment.

**Table 1:** Mean haemoglobin levels (g/dL) in mild and moderate iron deficiency anemia groups before and after treatment Abbreviations: Hb, hemoglobin; SD, standard deviation.

Iron deficiency anemia	Before treatment (mean Hb ± SD)	After treatment (mean Hb ± SD)	P-value
Group 1	6.2 ± 0.2	12.3 ± 0.13	< .001
Group 2	9.4 ± 0.72	13.5 ± 0.28	< .001

**Table 2:** Mean haemoglobin levels (g/dL) in mild and moderate vitamin B12 deficiency anemia groups before and after treatment Abbreviations: Hb, haemoglobin; SD, standard deviation.

Vitamin B12 deficiency anemia	Before treatment (mean Hb ± SD)	After treatment (mean Hb ± SD)	P-value
Group 1	6.7 ± 0.17	12.6 ± 0.18	< .001
Group 2	9.3 ± 0.32	13.2 ± 0.14	< .001

For iron-deficiency anemia patients, those in Group 1 showed a significant reduction in mean HbA1c from 6.1% ± 0.23% to 5.1% ± 0.14% (P < .01). Group 2 iron-deficiency patients showed a significant reduction of mean HbA1c from 5.5 ± 0.24% to 4.6 ± 0.2% (P < .001; Table 3).

**Table 3:** Mean HbA1c concentrations in mild and moderate iron-deficiency anemia groups before and after treatment Abbreviations: HbA1c, glycated haemoglobin; SD, standard deviation.

Iron deficiency anemia	Before treatment (mean HbA1c ± SD)	After treatment (mean HbA1c ± SD)	P-value
Group 1	6.1 ± 0.23	5.1 ± 0.14	< .001
Group 2	5.5 ± 0.24	4.6 ± 0.2	< .001

For vitamin B12-deficiency anemia patients in Group 1, the initial mean HbA1c was 5.9% ± 0.3%, and it decreased significantly to 5.0% ± 0.15% with treatment (P < .001). Group 2 B12-deficiency patients also showed a significant decrease in HbA1c with the treatment of anemia. The initial mean HbA1c in Group 2 were 5.6 ± 0.19%, and it decreased to 4.9 ± 0.16% with treatment (P < .001; Table 4).

**Table 4:** Mean HbA1c concentrations in mild and moderate vitamin B12 deficiency anemia groups before and after treatment Abbreviations: HbA1c, glycated haemoglobin; SD, standard deviation.

Vitamin B12 deficiency anemia	Before treatment (mean HbA1c ± SD)	After treatment (mean HbA1c ± SD)	P-value
Group 1	5.9 ± 0.3	5.0 ± 0.15	< .001
Group 2	5.6 ± 0.19	4.9 ± 0.16	< .001

Table 5 presents the difference between HbA1c concentrations before and after treatment in Group 1 and Group 2 of both types of anemia. Initially, the baseline increase in HbA1c in Group 1 is higher than Group 2. After treatment, there is a significant decrease in HbA1c in both groups. For every 1 g/dL rise in Hb, HbA1c fell by 0.16 ± 0.03% on average for iron deficiency anemia and 0.15 ± 0.03% on average for B12-deficiency anemia; however, the degree of anemia also affected HbA1c concentrations.

**Table 5:** The difference of HbA1c (%) values before and after treatment in group one versus group two of both types of anemia Abbreviation: HbA1c, glycated haemoglobin.

Nutritional anemia types	Group 1	Group 2	P-value
Iron deficiency anemia	6.1 ± 0.23	5.5 ± 0.24	< .001
Vitamin B12 deficiency anemia	5.9 ± 0.3	5.6 ± 0.19	< .001

## Discussion

We considered nutritional anemia’s effects on HbA1c, with a particular focus on iron and vitamin B12 deficiency anemia. We found that there was a significant decrease in HbA1c in both groups of iron deficiency patients after treatment. Similar results were obtained in the studies done by Coban et al. [13], Ford et al. [14], and El-Agouza et al. [15]. We found that HbA1c values were higher for patients with severe anemia when compared with patients with mild and moderate anemia. A study by Silva et al. obtained similar results [16].

Our findings contradicted those reported by Sinha et al., who reported a subsequent rise in HbA1c with iron supplementation (4.6% to 5.9%) in 50 iron deficiency anemia patients and 50 controls [2]. Similar contradictory results were also reported by Van Heyningen et al. [17].

The vitamin B12-deficiency anemia patients showed a significant decrease in HbA1c with treatment for anemia. Very few studies in the literature analyzed the relationship between B12 deficiency and HbA1c. Gram-Hansen et al. conducted a study that included both iron (n=10) and B12 (n=10) deficiency anemia patients [18]. They reported no significant differences in HbA1c concentrations in iron-deficient patients, vitamin B12 deficient patients, and controls. The study conducted by Capoor et al. [19] had results similar to our study. We found that HbA1c was higher in the vitamin B12 deficiency anemia subjects with severe anemia when compared with mild and moderate anemia subjects. According to Pani et al. [20] and Kilpatrick et al. [21], the age of the individuals also plays a vital role in the HbA1c of the individuals. In their study, HbA1c increased with the age of the individuals. We found that HbA1c concentrations did not always change with the degree of anemia. Table 6 gives a brief view regarding the results of some studies on HbA1c in anemia [2,13,16-18].

**Table 6:** Select studies regarding HbA1c in anemia based on their results Abbreviation: HbA1c, glycated haemoglobin.

Studies showing a decrease in HbA1c levels with the treatment of anemia	Studies showing no change in HbA1c levels	Studies showing an increase in HbA1c levels with the treatment of anemia
Coban et al. [13]	Heyningen et al. [17]	Sinha et al. [2]
Silva et al. [16]	Gram-Hansen et al. [18]	

Limitations

A more extensive multicentric epidemiological study is needed to highlight the generalized burden (i.e., the effect of anemia on HbA1c levels in individuals without diabetes). As the present study was hospital-based, our findings should not be generalized to the whole population. Other factors that alter HbA1c values in a general population should also be considered.

## Conclusions

HbA1c in both types of anemia subjects showed a significant reduction with appropriate anemia therapy. The considerable variations in HbA1c in anemia patients might contribute to inaccurate diabetes status classification of these subjects. There is an observable, albeit limited, inverse relationship between rising Hb levels and falling HbA1c levels in patients with anemia. Hence anemia should be treated before using HbA1c as a diagnostic tool for diabetes. Physicians should consider confirming Hb values before determining the glycemic status of anemic patients as this coexistence may lead to misinterpretation of diabetes status.
